# Paracrine factors from adipose-mesenchymal stem cells enhance metastatic capacity through Wnt signaling pathway in a colon cancer cell co-culture model

**DOI:** 10.1186/s12935-015-0198-9

**Published:** 2015-04-18

**Authors:** Dongmei Chen, Shudan Liu, Huiming Ma, Xueyun Liang, Haibin Ma, Xiurui Yan, Bao Yang, Jun Wei, Xiaoming Liu

**Affiliations:** Institute of Human Stem Cell Research, the General Hospital of Ningxia Medical University, Yinchuan, Ningxia, 750004 China; Key Laboratory of Fertility Preservation and Maintenance of Ministry of Education, Ningxia Medical University, Yinchuan, Ningxia, 750004 China; Department of Colorectal Surgery, the General Hospital of Ningxia Medical University, Yinchuan, 750004 China; Key Laboratory of Ministry of Education for Conservation and Utilization of Special Biological Resources in Western China, Ningxia University, Yinchuan, Ningxia, 750021 China

**Keywords:** Adipose mesenchymal stem cells, Colon cancer, Wnt, Paracrine factor, Malignancy

## Abstract

**Background:**

Mesenchymal stem cells (MSCs) in tumors have emerged as progenitors involved in stroma formation and metastasis of cancers, partially owing to their abilities to differentially express paracrine factors related to the proliferation and invasion of cancer cells. In this regard, increasing evidence has shown that MSCs have impacts on the malignancy of colon cancer, however, the underpinning mechanisms by which MSCs promote cancer metastasis remain elusive.

**Methods:**

To investigate the crosstalk between adipose-derived MSCs (AMSCs) isolated from adipose tissues and colon cancer cells, a co-culture transwell model of AMSCs and colon cancer cells was employed, and the activation of Wnt signaling and paracrine factors in colon cancer cells and AMSCs were measured.

**Results:**

The results showed that AMSCs could enhance the metastatic capacity of colon cancer cells with an elevated expression of mesenchymal-epithelial transition (EMT)-associated genes in a contact-dependent manner. Reciprocally, colon cancer cells were able to induce AMSCs to produce metastasis-related factors and cytokines, such as FGF10, VEGFC and matrix metalloproteinases (MMPs) in part through a mechanism of an activation of Wnt signaling, by which these factors in turn activate Wnt signaling of colon cancer cells. Intriguingly, an inhibition of Wnt signaling leads a reduced capacity of invasion and colony formation of colon cancer cells *in vitro*, and the tumorigenicity of cancer cells in a murine model.

**Conclusions:**

These findings thus suggest that the crosstalk between the Wnt signaling of cancer cells and paracrine factors of AMSCs has an implication in colon cancer malignancy. This study thus uncovers a novel Wnt-paracrine factors mediated-crosstalk between colon cancer cells and AMSCs in cancer malignancy.

## Background

The tumor and its microenvironment are closely related and constantly interacted. The tumor microenvironment constitutes to tumor cells within the local environment such as local infiltration of immune cells from the tumor, mesenchymal cells and cytokine networks. It is well established that the tumor microenvironment plays a critical role in the development and metastasis of cancers. Accumulating studies have evidenced that tumor-associated mesenchymal stem cells (MSCs) are involved in the acceleration of cancer progression and able to promote invasion and migration of cancer cells. *Vice versa*, however, cancer cells are also able to actively modulate nonmalignant stromal cells of tumors, including mesenchymal cells [1]. The most abundant stromal cells in the tumor microenvironments are tumor-associated fibroblasts (TAFs), which are irritated by cancer cells and associated with poor prognosis in cancer patients [2]. TAFs display an alternatively activated phenotype that can directly affect cancer cell growth, neoangiogenesis and extracellular matrix remodeling [3]. However, whether such a modulating ability of cancer cells in tumor microenvironments is cancer-type dependent, and the precise cellular and molecular mechanism of interactions between cancer cells and TAFs undergoing a malignancy remains unclear.

Mesenchymal stem cells (MSCs) found in many adult tissues are multipotent progenitor cells that possess a capacity of self-renewal and multilineage differentiation [4], and properties of immunomodulation [5]. MSCs have been proposed as an attractive candidate for the delivery of anti-tumor agents, owing to their ability to home into tumor sites and to secrete cytokines [6]. Despite recent findings suggest that the MSC-secreted soluble factors in a tumor microenvironment may contribute more significantly in tumor progression [7,8], the underlying mechanisms of MSCs in the process of tumor progression are much less understood, and there are controversial reports suggest both of tumor-promoting and tumor-inhibiting roles of MSCs.

Obesity has been associated with numerous negative health effects including several types of cancer. Fat tissue is a rich source of both endothelial progenitors and multipotent MSCs. Adipose MSC (AMSC) phenotypically resemble bone marrow MSCs (BMSCs) and share their characteristics of multipotency. There are investigations show that AMSCs can play a positive role in tumor proliferation and malignancy through multiple signaling pathways, by which they play a pivotal role in the regulation of cancer growth by modulating Akt activity and pathways involving in EGFRs, FGFRs, ERbB2 receptor-mediated pathways [9-11]. In addition, AMSCs are more likely recruited into tumors, and function to increase tumor vascularization by endothelial translation, and elevate proliferation of neighboring malignant cells, as compared with BMSCs [12]. Zhang *et al.* shown that a recruitment of adipose stromal cells by tumors was sufficient to promote tumor growth [13]. Therefore, there is a necessity to understand the cell-cell communication between the AMSCs and the cancer cells of tumor, which may allow us to uncover sequential events that lead to cancer progression and develop novel agents for anticancer therapy.

Emerging evidence suggests that multiple cellular elements in the tumor microenvironment are co-evolved during the process of carcinogenesis. Bi-directional paracrine signals coordinately regulate tumorigenic cell populations and surrounding cells including MSCs [14,15], by which tumorigenic cells can produce factors to attract and regulate a variety of cell types that constitute the tumor microenvironment. For example, GRP78 secreted by tumor cells can stimulate the differentiation of BMSC to cancer-associated fibroblasts [16]. Interestingly, many of the pathways activated during tumor formation resemble a cross networks, including cytokine loops and transcriptional factors [1]. There findings support the notion of that cancer cells are able to induce AMSCs to produce paracrine molecules, which in turn promotes the malignancy of cancer cells.

Stem cell regulatory signaling including the Notch, Hedgehog, Wnt, PI3K, NF-κB, and Jak/STAT pathways are frequently dysregulated in tumor cells. These pathways are activated in some tumors by mutation of key regulatory elements. For instance, a dysregulation of Wnt signaling often occurs in colon cancer, in which the Wnt signaling is hyperactiviated, since an APC mutation is always found in this type of cancer [17,18]. Thus, it has been suggested that the hyperactivated Wnt signaling may ultimately resulting in an enhanced transcription of specific genes in the stroma cells of microenvironment of colon tumor, which in turn promotes the metastasis of colon cancer [19]. However, the mechanism underpinning the coordination of cancer cells and AMSCs of tumor microenvironment in colon cancer metastasis remains unclear.

In the present study, we sought to identify potential protein associated with colon cancer malignancy instigated by prometastatic MSCs using a co-culture cell model. We found that AMSCs could endow colon cancer cells with enhanced tumor-initiating capability and metastatic traits in a contact dependent manner, when the cancer cells were cultured with AMSCs in comparison with that cultured in AMSC condition medium alone. The Wnt3a secreted by colon cancer cells could activate Wnt signaling in AMSCs and induce AMSCs to trigger the secretion of a select set of proteins, converge on and increase the expression of the stemness transcriptional factors and EMT-associated genes.

## Materials

### Ethics statement

Human adipose tissue was collected with a protocol approved by the Ethic Committee for the Conduct of Human Research at Ningxia Medical University. Written consent was obtained from every individual according to the Ethic Committee for the Conduct of Human Research protocol. All participants were provided written informed consent for the publication of the data. The Human Research Ethic Committee at Ningxia Medical University approved this study.

### Animals and chemicals

Severe combined immunodeficiency (SCID) mice were obtained from Vital River Laboratories (VRL). All animal study was performed with a protocol approved by the committee of animal care and use at the Ningxia Medical University. All chemical reagents used in this study were products of Sigma-Aldrich (St Louis, MO, USA), unless otherwise indicated.

### Cell cultures

AMSCs were isolated from human adipose tissue of patient undergone abdominal surgery at the Department of Surgery in the General Hospital of Ningxia Medical University. All adipose tissues were resected from tissues >10 cm away from tumor sites. The adipose tissue was immediately digested with 1 mg/ml collagenase A (Roche Diagnostic) in Dulbecco’s modified essential medium F12 (DMEM:F12, 1:1 Gibco) for 60 min at 37°C. The dissociated tissue was the filtered through a 70 μm nylon membrane to remove the indigested mass of tissue. The cell suspension was then centrifuged at 300 g for 10 min, and the cell pellet was resuspened with red blood lysis buffer (155 mmol/l NH_4_Cl, 20 mmol/l Tris pH 7.6) to remove red blood cells. The cells were pelleted by centrifugation and resuspended in DMEM-F12 (1:1) medium supplemented with 15% fetal bovine serum (FBS), 10 ng/ml of bFGF, 1% insulin-transferrin-selenium (ITS) (Gibco), 1% pen-strep (Gibco). The collected cells were the fraction of stroma cells of the adipose tissue. The isolated stroma cells were then seeded in plastic plates at the initial density of 3.2 × 10^4^ cells/cm^2^ non-adherent cells were removed by changing the culture medium. The cells were refreshed twice a week. Colon cancer cell lines, HCT116 cells (ATCC # CCL-247), LoVo cells (ATCC # CCL-229), SW480 (ATCC # CCL-228), LS174T (ATCC # CL-188), CCD-18Co (ATCC # CRL-1459) were grown in Dulbecco’s modified essential medium with 10% FBS.

### Co-culture system

In order to investigate functions of AMSCs in the progression of colon cancer, colon cancer HCT116 cells were either cultured in a cell co-culturing model, in which HCT116 cells were cultured on transwell of 0.4 μm pore size membrane (up chamber) and AMSCs were cultured on the bottom chamber of the culturing well, or cultured with conditional medium of human AMSCs (AMSC-CM). To prepare AMSC-CM, a confluent AMSC culture was refreshed with serum-free basic medium and cultured for additional for 24 h, the supernatant of culture was then collected as basic conditioned medium for preparation of AMSC-CM.

### Flow cytometry assay

The Flow cytometry assay was conduced by using AMSCs cultured at passage three (P3). Following the culturing, the cells were harvested and extensively washed prior to be labeled with fluorescent-conjugated (PE or FITC) antibodies to CD105, CD90, CD73, CD45, CD116 and CD34. The flow cytometry analysis was performed on BD FACSCalibur™ platform.

### ELISA assay

AMSCs were seeded alone or separated from transwell apparatus that co-cultured with HCT116 and refreshed with regular culture medium for additional 24 h. The culture media were then collected and used for accessing the secretion of paracrine factors (FGF10, VEGFC, TNFα and IL10) by ELISA using commercial available kits (eBiosciences, CA). The number of AMSCs was also counted for the subsequent assessment.

### Immunohistochemistry and immunofluorescence staining

The cells were fixed in 4% paraformaldehyde (PFA) at room temperature (RT) for 30 min, and permeabilized with 0.1% Triton X-100 in PBS for 10 min. The slides were then blocked with 3% BSA (Sigma) in PBS at RT for 1 h, followed by incubating with the primary antibody to vimentin (1:100, SantaCruz, USA) or E-cadherin (1:100, SantaCruz, USA) at 4° overnight. Cy3-labelled anti-rabbit-IgG secondary antibody was used for visualization of specific signal under a fluorescent microscope. The nuclei of cells were count stained with DAPI-Fluoromount-G.

### MTT assay

An MTT (methyl thiazoly tetrazolium) assay was employed to ascertain the cell viability. HCT116 cells were seeded at densities 2000 cells per well in 96-well plate. The proliferation of cells was measured by an MTT assay daily from day 2 to day 7 of culturing. Briefly, 20 μL of 5 mg/mL MTT (Sigma) solution was added to each well and incubated at 37°C for 4 h, after which the medium was replaced by 100 μL of DMSO (Sigma) and incubated for additional 10 min. The capacity of cell proliferation was then determined by Microplate spectrophotometer at 560 nm (Bio-Rad Laboratories Inc., Hercules, USA). Data represent the mean of six wells for each point.

### Colony formation assay

HCT116 cells were seeded at a density of 1 × 10^5^/well into 60 mm culture dishes, and cultured with DMEM containing 0.5% of FBS at 37°C for two weeks. The cultures were washed twice with PBS prior to be stained with 0.1% crystal violet solution. The number of colonies containing >50cells was counted under a microscope. The experiments were performed in triplicate.

### Transwell invasion assay

The apical surface of Transwell membrane (8 μm pore size, BD Bioscience) was coated with Matrigel (BD Bioscience). 40 μl of Metrigel (1:5 matrigel: DMEM) was applied onto each of the upper chamber of 12 mm Transwell insert. The insert was then incubated at 37°C for 4–5 h for gel formation. 5 × 10^4^ HCT116 cells from different groups were suspended in 200 μl of 0.5% serum medium and seeded on the top of gel, and 500 μl of medium supplemented with 10% FBS were added to the lower compartment of culture chamber. After 24 h of incubation, the cultures were fixed with 4% paraformaldehyde, and stained with 0.1% crystal violet for determining cells migrated to the basolateral surface of the membrane under a light microscope. All experiments were performed in triplicate.

### Quantitative real-time polymerase chain reaction (qRT-PCR)

Total RNA was extracted using miRNeasy kit (QIAGEN) and was processed for reverse transcription (RT) with a RT kit (Thermo) per manufacturers’ instructions. The quantitative PCR was carried out on an IQ5 thermal cycler (BIO-RAD, USA) and Maxima® SYBR Green/ROX qPCR Master Mix (Thermo) using gene specific primers (Table 1).

**Table 1:** **Primers and probes used for qPCR assays**

**Gene**	**Sequence 5′ → 3′**	**Gene**	**Sequence 5′ → 3′**
ZEB1	F: TTGGTTTGGTGTCTCCCATAAGT	IL-10	F: GAGAACCAAGACCCAGACATCAAG
	R: ACATTACCATCTACCGCCACTTTA		R: GGCATTCTTCACCTGCTCCAC
Snail	F: GACCCCAATCGGAAGCCTAA	LIF	F: CAGTGCCAATGCCCTCTTTATT
	R: TGGTCGTAGGGCTGCTGGA		R: GGAGGTGCCAAGGTACACGACT
Slug	F: GAAGAGGAAAGACTACAGTCCAAGC	INFγ	F: TCAGATGTAGCGGATAATGGAACT
	R: CCAGGCTCACATATTCCTTGTCA		R: CATTCATGTCTTCCTTGATGGTCT
Twist1	F: GCAAGAAGTCTGCGGGCTG	IL1β	F: TTATTACAGTGGCAATGAGGATGAC
	R: AACGCCTCGTTCAGCGACT		R: TGCTGTAGTGGTGGTCGGAGA
E-cadherin	F: AAGGCAAGGTTTTCTACAGCATC	TNFα	F: GAGTGACAAGCCTGTAGCCCAT
	R: CCATTGGATCCTCAACTGCATT		R: CCTTGAAGAGGACCTGGGAGT
Oct-4	F: CTTGCTGCAGAAGTGGGTGGAGGAA	TGFβ	F: TGCCCCGAGTGCTACTTTGA
	R: CTGCAGTGTGGGTTTCGGGCA		R: GCAACTGACGCAGCAGAAATG
Nanog	F: CAGAAGGCCTCAGCACCTAC	MMP1	F: TGAAGAATGATGGGAGGCAAGTT
	R: ATTGTTCCAGGTCTGGTTGC		R: AGGGTTTCAGCATCTGGTTTCC
Sox2	F: AGCTACAGCATGATGCAGGA	MMP2	F: GACAGTGGATGATGCCTTTGCT
	R: GGTCATGGAGTTGTACTGCA		R: GGAGTCCGTCCTTACCGTCAA
Bmi1	F: CCTCATCCACAGTTTCCTCACAT	MMP9	F: TCCACCCTTGTGCTCTTCCCT
	R: TATTGGCAAAAGAAGATTGGTGG		R: CTGCCACCCGAGTGTAACCAT
C-MYC	F: ATACATCCTGTCCGTCCAAGCA	MMP11	F: AGTGCCCGCAACCGACAG
	R: ACAAGAGTTCCGTAGCTGTTCAAG		R: GGCGTCACATCGCTCCATAC
VEGFA	F: TGTGAATGCAGACCAAAGAAAGA	ICAM1	F: GTGCTATTCAAACTGCCCTGATG
	R: CAGGGAACGCTCCAGGACTTAT		R: CTGGCAGCGTAGGGTAAGGTTC
VEGFC	F: TCTGGAGGAGCAGTTACGGTCT	COL1A1	F: CAAGACGAAGACATCCCACCAAT
	R: GTTATGTTGCCAGCCTCCTTTC		R: ACAGATCACGTCATCGCACAAC
VEGFD	F: ATCGGTCCACTAGGTTTGCG	COL6A1	F: GAGACGATAACAACGACATTGCA
	R: AACAGCCACCACATCGGAAC		R: GATGATGTCCAAAATCTCGCATT
FGF2	F: TACCTGGCTATGAAGGAAGATGG	β-catenin	F: GTGGTATAGAGGCTCTTGTGCG
	R: AGTTCGTTTCAGTGCCACATACC		R: TCCAACAGTAGCCTTTATCAGAGG
FGF10	F: TAACTGGCAGCATAATGGGAGG	Wnt3a	F: CGGTGACTTCCTCAAGGACAAG
	R: CCATTGGAAGAAAGTGAGCAGAG		R: AGGAGCCCGTCTCAGGGTT
HGF	F: ATTGCCCTATTTCTCGTTGTG	CD44	F: ATCACCGACAGCACAGACAGAATC
	R: GCATTTCTCATCTCCTCTTCC		R: GAAACAACCATGAAAACCAATCCC
IGF	F: CGCTGTGCCTGCTCACCTTC	CyclinD1	F: TGTCGCTGGAGCCCGTG
	R: GCCATACCCTGTGGGCTTGT		R: GGATGGAGTTGTCGGTGTAGATG
IL6	F: AATGAGGAGACTTGCCTGGTGA	ZEB2	F: TCCCTTCTGCGACATAAATACG
	R: GGTTGGGTCAGGGGTGGTTAT		R: CATGTGCTGCGAGTACGAGC
IL8	F: CTCTTGGCAGCCTTCCTGATTT	GAPDH	F: GTGGACCTGACCTGCCGTCT
	R: GGTGGAAAGGTTTGGAGTATGTCTT		R: GGAGGAGTGGGTGTCGCTGT

### Western blot analysis

Cells were lysed with RIPA buffer (Thermo Scientific, USA) included DTT, PMSF, protease inhibitor cocktail and phosphatase inhibitor cocktail (Active Motif, USA) and the protein concentration was determined using Pierce BCA protein Assay kit per the manufacturer’s instruction (Thermo Scientific, USA). 20 μg of protein was resolved using a 10% SDS-PAGE Gel prior to be transferred on PVDF membrane (Life Technologies, USA). The membranes were blocked with 5% BSA for 1 h and probed with respective primary antibodies overnight such as Anti-phospho-β Catenin (Ser37) (Millipore, Cat. No. NG1664798), GSK-3β (Transduction Laboratories Cat. No. G22320), anti-phos-GSK3β (Millipore, Cat. No. 05–413), and Anti-Active-β-Catenin (Anti-ABC) Antibody (Millipore, Cat. No. 05–665), followed by incubating with appropriate secondary antibody conjugated with HRP (Santa Cruz Biotechnology, USA) at RT for 1 h. The interest proteins were detected by ECL kit (Santa Cruz Biotechnology, USA). Blot images were captured using ChemiDoc MP (BIO-RAD, USA). Densitometric analysis was performed using ImageJ software and values were normalized with loading control (GAPDH).

### *In vivo* analysis of tumorigenetic capacity

The tumorigenic capacity of HCT116 co-cultured with AMSCs was evaluated in 6–8 week-old of female SCID mice. The mice were subcutaneously inoculated (s.c.) with 200 μL Matrigel (BD Bioscience) containing 5 × 10^6^ of cancer cells. The animals were euthanized and the tissues of injection sites were collected for analysis of tumorigenicity eight weeks after the injection. The harvested tissues were then used for histological and proliferation analysis by HE (hematoxylin and eosin) histochemistry staining and immunohistochemistry (IHC) staining using anti-Vementin and E-cadherin antibodies (Santa Cruz biotect, Santa cruz, CA, USA), respectively. 9 SCID mice were analyzed for each group.

### Statistical analysis

All statistical analyses were performed using SPSS statistical analysis software (SPSS Inc., Chicago, IL, USA). The results were presented as the mean ± standard deviation (SD) for comparison of individual conditions, and differences between the groups were tested by one-way ANOVA with a post-hocleast square difference (LSD) test. A p < 0.05 was defined as a statistical significance.

## Results

### AMSCs enhance colon cancer stem cell-like property

**Figure 1:**
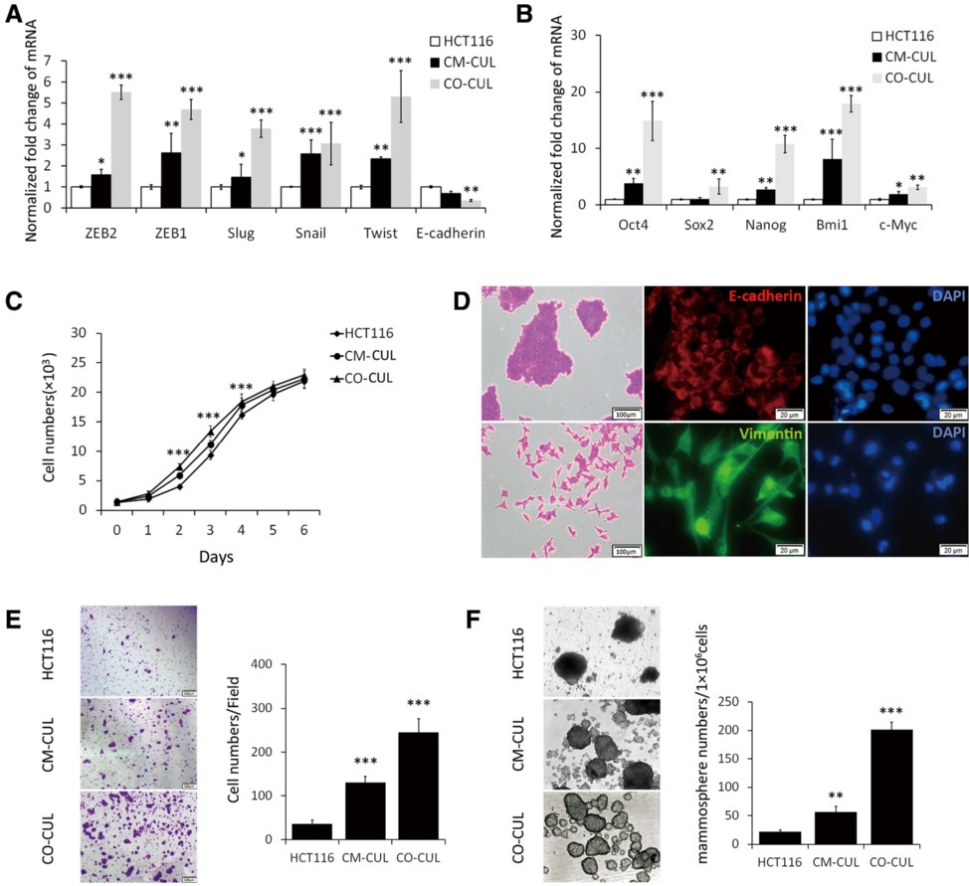
AMSCs enhance malignancy of colon cancer cells in a transwell co-culture model. Colon cancer cells were co-cultured with AMSCs using a transwell model. The malignancy of HCT116 cells was ascertained by examining the expression of EMT- and stemness-related genes, and capacities of proliferation, invasion and colony formation. **(A)** The transcripts of EMT-associated genes in HCT116 treated with indicated conditions were determined by a qRT-PCR assay. **(B)** The transcripts of stemness-related genes in HCT116 treated with indicated conditions were determined by a qRT-PCR assay. **(C)** The proliferation of HCT116 cells cultured in the indicated conditions was analyzed by an MTT assay. **(D)** Immunofluorescent analysis of E-cadherin and vimentin proteins in HCT116 cells cultured in regular medium or in the co-culture system as described in **(A)**. The scale bar represents 20 μm. **(E)** The capacity of invasion of HCT116 cells in the indicated conditions was detected by a transwell invasive assay. Left panel: representative images of indicated condition; right panel: numbers of invaded cells in indicated conditions. **(F)** The capacity of colony formation of HCT116 cells in the indicated conditions was detected by a sphere formation assay. Left panel: representative images of indicated condition; right panel: numbers of sphered cells in indicated conditions. Data represented as mean ± SD from three independent triplicated experiments (N = 9). Compared to the control, *p < 0.05; **p < 0.01; ***p < 0.001.

In order to seek whether AMSCs have an impact on colon cancer cells, colon cancer HCT116 cells were primed with human AMSCs using either an *in vitro* co-culture model (co-cul) or AMSC-CM (cm-cul), and the alteration of expression of stem cell markers and EMT markers was evaluated by a qRT-PCR assay. Results revealed that the expression of EMT-associated genes *ZEB1*, *ZEB2, Slug*, *Snail* and *Twist* (Figure 1A), and “stemness” genes *Oct4*, *Sox2*, *Nanog*, *Bmi1* (Figure 1B) was markedly up-regulated in both conditions of co-cul and cm-cul, as compared with the untreated control HCT116 cells. Of note, the EMT-related gene, *E-cadherin* was significantly down-regulated (Figure 1A). More importantly, more robust expression of these genes was observed in HCT116 cultured in the co-culture model relative to the cells cultured using AMSC-CM. The proliferating ability of colon cancer cells was also enhanced in a co-culture condition as determined by an MTT assay (Figure 1C). Equally noteworthy, the malignant potential of HCT116 cells cultured in the co-culture model or with AMSC-CM was dramatically enhanced as compared to the controls (Figure 1D-F). Morphologically, HCT116 cells were altered from a rounded shape to an elongated one, morphology of cell with capacity of EMT, at day 7 following the co-culture, albert the expression of E-cadherin and vimentin proteins was detected in HCT116 cells (Figure 1D). Such an AMSC-enhanced migratory capacity of HCT116 cells was further supported by the results of transwell invasion assay (Figure 1E) and colony formation assay (Figure 1F), in which HCT116 cells co-cultured with AMSCs or cultured with AMSC-CM showed an enhanced ability of invasion (Figure 1E) and colony formation (Figure 1F), as compared with cells cultured in regular medium. These observations suggested that AMSCs could enhance the malignant capacity of HCT116 cells, implying that AMSCs may play a role in promoting tumor-associated phenotypes of colon cancer cells.

### Colon cancer cells enhance paracrine signaling of AMSCs

**Figure 2:**
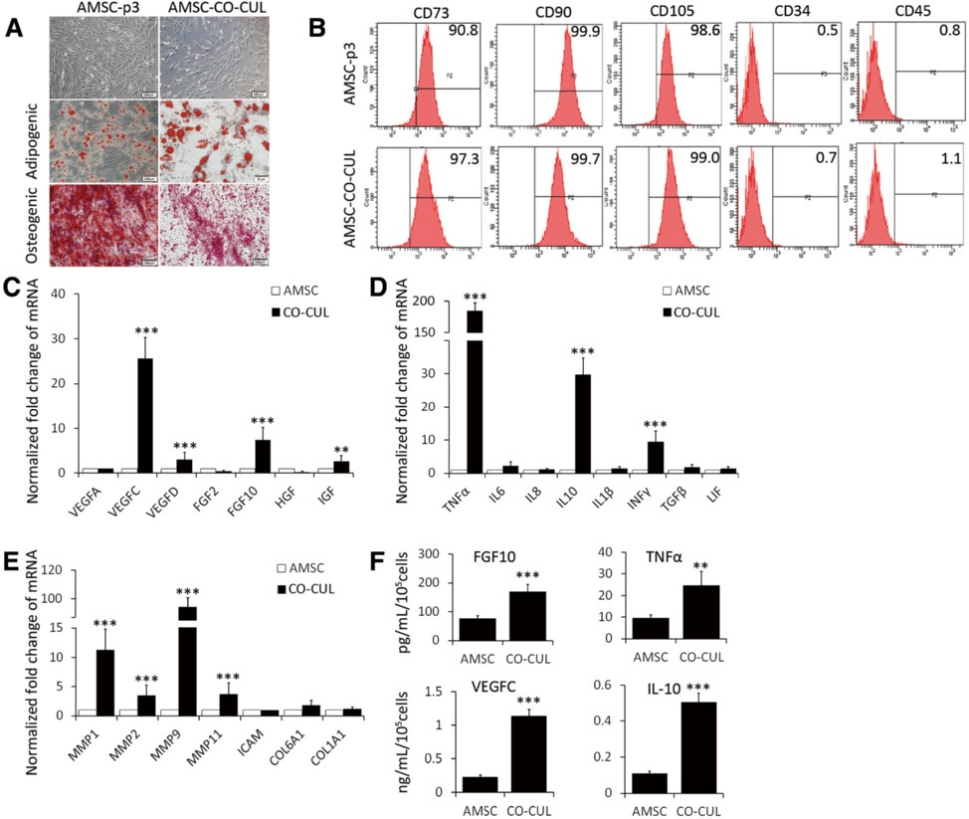
HCT116 cells alter the expression of paracrine factors in AMSCs of co-culture model. P3 AMSCs were co-cultured with HCT116 cells in a transwell model. The characteristics and differentiate properties, and abilities of the expression of paracrine factors and cytokines in the co-cultured P3 AMSCs were examined. **(A)** The morphology and differentiation of original P3 AMSCs and AMSCs in the co-cultured model. AMSCs showed a normal MSC morphology and potency of osteogenic and adipogenic differentiation. The scale bar represents 20 μm. **(B)** Flow cytometry analysis for MSC-specific phenotype of AMSCs in the co-culture model. The HCT116 co-cultured P3 AMSCs retained a normal AMSC immunophenotype. **(C-E)** The alteration of expressions of indicated paracrine factors **(C)**, cytokines **(D)** and EMT-associated genes in AMSCs was determined by a qRT-PCR assay. **(F)** Concentration of indicated paracrine factors and cytokines of the culture medium was ascertained by an ELISA. Data represented as mean ± SD from three independent triplicated experiments (N = 9). Compared to the control, *p < 0.05; **p < 0.01; ***p < 0.001.

We next sought to whether colon cancer cells were able to alter the property of AMSCs. The cell surface phenotype and multipotent differentiation potential of AMSCs after co-culturing with colon cancer cells were first explored. To evaluate the differentiation potential of AMSCs after co-culture with HCT116 cells, the AMSCs were cultured with induction medium for adipogenesis or osteogenesis. As shown in Figure 2A, oil red O staining showed accumulated triacylglycerol staining of adipogenesis, and Alizarin red S staining showed mineral depositions of osteogenesis in the cell cultures. Flow cytometry analysis using the co-cultured AMSCs demonstrated that over 90% of AMSCs remained their ability to express MSC surface immunophenotypic markers, such as CD105, CD73 and CD90, but lack expression of hematopoietic markers CD34 and CD45 (Figure 2B). These data indicated that the characters of AMSCs were unaltered after they were co-cultured with HCT116 cancer cells, as compared with the original P3 AMSCs (Figure 2A, B). Intriguingly, in the culture of AMSCs contacted with HCT116 cells showed an elevated abundance of transcripts of growth factors of *VEGFC* and *FGF10* (Figure 2C), cytokines of *TNF*α, *IL10* and *INF*γ (Figure 2D), and extracellular matrix *MMP1*, *MMP2*, and *MMP11* (Figure 2E). In addition, the increased expression of several above genes was further validated at protein level by ELISA, by which more abundant proteins of VEGFC, FGF10, TNFα and IL10 were detected in AMSCs co-cultured with HCT116 cells (Figure 2F). These results suggest that colon cancer cells may enhance paracrine signaling of AMSCs within a cancer microenvironment.

### Colon cancer cells activate Wnt signaling in AMSCs

**Figure 3:**
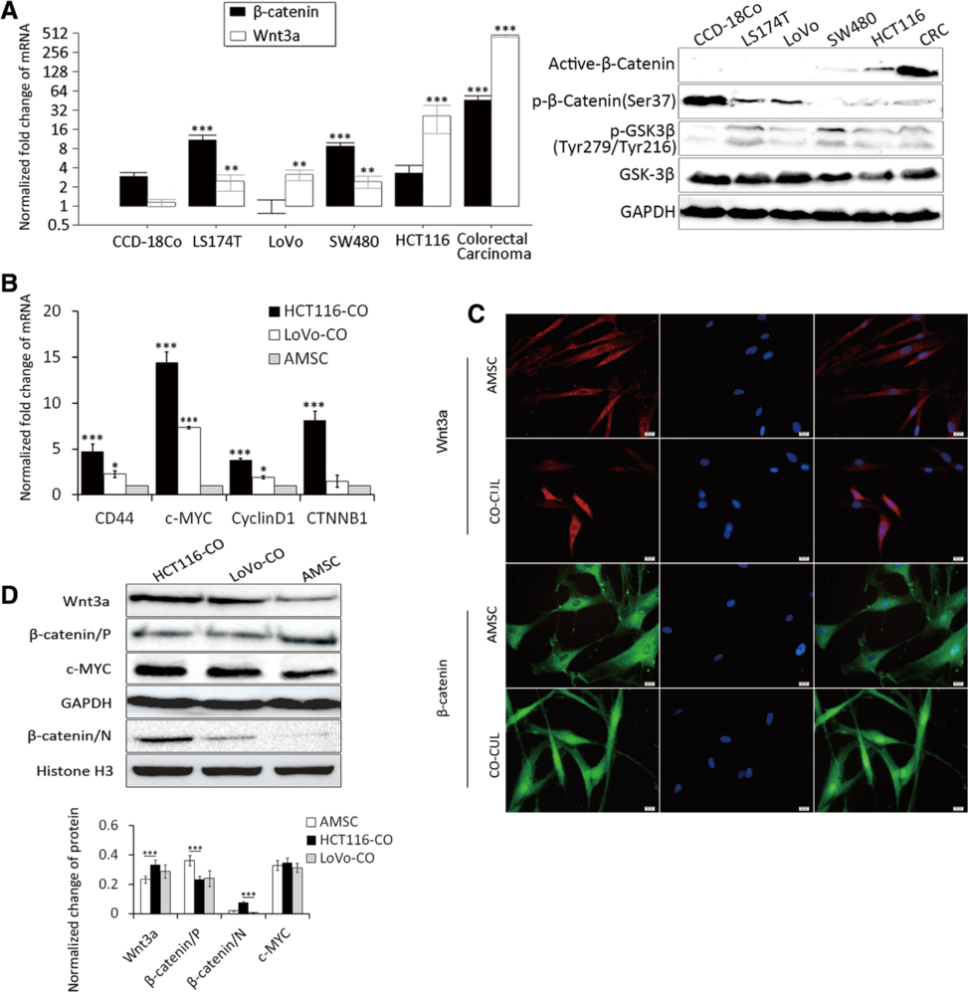
The activated canonical Wnt signaling of colon cancer cells induces TAM phenotypes of AMSCs in co-culture model. The expression of Wnt signaling molecules and its target genes in HCT116 and AMSCs of co-culture model was detected for examining Wnt activation in these cells. **(A, B)** The alteration of transcripts of Wnt3a and beta-catenin as well as protein expressions of actived actived β-catenin, phosphorylated (Ser37) β-Catenin and phosphorylated (Tyr279/Tyr216) GSK3β **(A)**, and indicated Wnt target genes of different colon cancer cells or in co-culture models **(B)** was determined by a qRT-PCR assay. **(C)** Representative images of confocal fluorescent microscopy of immunofluorescent staining for Wnt3a and beta-catenin of AMSCs cells cultured in indicated conditions. The scale bar represents 20 μm. **(D)** Immunoblotting assay for indicated Wnt signaling and its target genes in cells cultured in an indicated conditions. GAPDH and Histone H3 in A severed as loading controls for cytosolic and nuclear proteins, respectively. Top panel: immunoblots of indicated proteins; bottom panel: fold changes of interest proteins in top panel quantified by a densitometry analysis. Compared to the control, *p < 0.05; **p < 0.01; ***p < 0.001.

In order to explore which developmental signaling pathway(s) of colon cancer cells are involved in the up-regulation of metastasis-related genes in AMSCs, the role of canonical Wnt signaling in the regulation of paracrine activity of colon cancer cells was interrogated. The baseline activities of canonical Wnt signaling in colon cancer cell lines and colorectal carcinoma (CRC) tissues were examined by accessing the expression of Wnt3a and β-catenin. More abundant β-catenin and/or Wnt3a transcripts were observed in colon cancer LS174T, SW480 and HCT116 cells and CRC tissues, but not LoVo cells as compared with the normal colonic epithelial cell line CCD-18Co (Figure 3A). However, in comparison with normal epithelial CCD-18Co cells, all colon cancer cells and CRC tumor tissues exhibited less phosphorylated β-catenin proteins and more phosphorylated GSK3β proteins, indicative of activated Wnt/β-catenin signaling. In addition to CRC tissues, HCT116 cells also showed an increased expression of active β-catenin protein and a less abundant phosphorylated (Ser37) β-Catenin by inhibition of GSK3β (Figure 3A) relative to other tested cancer cell lines. In contrast, LoVo cells displayed the least abundant β-catenin transcripts and β-catenin activity among the tested colon cancer cell lines (Figure 3A). HCT116 and LoVo cells were thus used in further studies in co-culture model. AMSCs co-cultured with HCT116 cells or LoVo cells exhibited marked increases of the expression of Wnt target genes, including *CD44*, *c-MYC*, *CyclinD*1, and *CTNNB1* as determined by a qRT-PCR assay (Figure 3B). Immunofluorescent staining further revealed that Wnt3a and beta-catenin proteins were elevated in AMSCs when they were co-cultured with HCT116 cells (Figure 3C), indicative of an activation of canonical Wnt pathway. Furthermore, immunoblotting assay revealed that the nuclear beta-catenin protein was significantly increased in AMSCs co-cultured with HCT116 cells, despite AMSCs co-cultured with LoVo cells only showed a decent increase of nuclear beta-catenin expression, in comparison with the control AMSCs (Figure 3D). Together, these observations suggest that Wnt signaling pathway may play a unique role in activation of both VEGFC and FGF10 signaling, highlighting that an activation of Wnt signaling may be a potential common effect for AMSCs in response to Wnt-activated cancer cells in multiple settings.

### The effect of AMSCs-induced EMT on stem cell-like behavior through canonical Wnt signaling pathway

**Figure 4:**
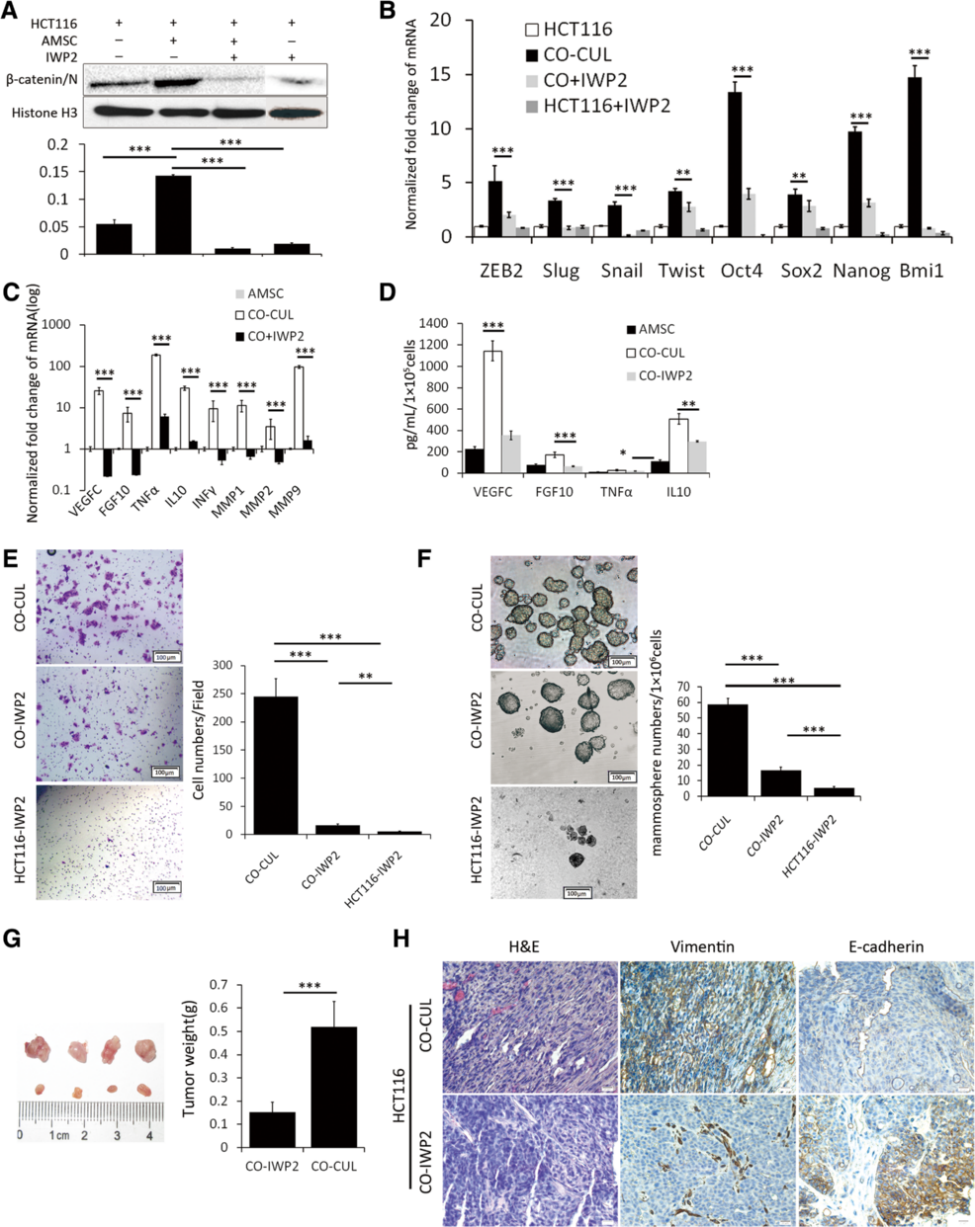
The Wnt signaling plays a key role in the production of paracrine factors of AMSCs malignancy of colon cancer cells in co-culture model. HCT116 cells were co-cultured with AMSCs in the presence of Wnt signaling inhibitor IWP2, and the production of paracrine factors of AMSCs and the malignancy of HCT116 cells were investigated *in vitro* and/or *in vivo* assays. **(A)** Representative immunoblots of nuclear beta-catenin from HCT116 cells cultured in indicated conditions (top panel) and their fold changes quantified by a densitometry analysis (bottom panel). **(B-C)** The alterations of transcripts of indicated EMT- and stemness-related genes of HCT116 cells **(B)**, and indicated cytokines and MMPs genes of AMSCs in different conditions **(C)**. **(D)** The concentration of indicated paracrine factors in the culture medium. **(E)** The capacity of invasion of HCT116 cells in the indicated conditions. Left panel: representative images of indicated condition; right panel: numbers of invaded cells in indicated conditions. **(F)** The capacity of colony formation of HCT116 cells in the indicated. Left panel: representative images of indicated condition; right panel: numbers of sphered cells in indicated conditions. **(G)** The *in vivo* tumorigenitic assay showed a repression of tumorigenicity of HCT116 cells in co-culture model with IWP2 as ascertained by the size (left panel) and weight (right panel) of subcutaneous primary tumors derived from SCID mice at 8 weeks after the injection of HCT116 cells. **(H)** Representative H&E and IHC staining of E-cadherin and vimentin in the primary tumors derived from SCID mice in **(G)**. Scale bars represent 20 μm. GAPDH was used as an endogenous control for normalization. Data represented as mean ± SD from three independent triplicated experiments (N = 9). Compared to the control, *p < 0.05; **p < 0.01; ***p < 0.001.

Since Wnt-excretive cancer cells activate AMSCs that can in turn induce CSC-traits of cancer cells, we investigated the impact of Wnt signaling of cancer cells on AMSCs. Wnt signaling inhibitor IWP-2 was employed to as an antagonist of the Wnt/β-catenin pathway by blocking Wnt secretion and activity. Western blotting assay showed that an addition of IWP2 led a decreased accumulation of nuclear β-catenin in HCT116 co-culture with and without AMSCs (Figure 4A), expression of stemness genes *(Slu*g, *Snail*, *Twist*, *Oct4*, *Sox2*, *Nanog*, *Bmi1*) (Figure 4B) and the EMT related genes (*ZEB1, ZEB2, FGF10*, *VEGFC*, *TNF*α, *IL10* and *MMPs*) (Figure 4C) in AMSCs co-cultured with HCT116 cells. In addition, the presence of IWP-2 could dramatically inhibit the secretion of FGF10 and VEGFC protein in the AMSC culturing medium (Figure 4D). The capacities of invasion (Figure 4E) and colony formation (Figure 4F) of cancer cells were also reduced when the IWP2 was added in the co-culture system, while an addition of IWP2 resulted in a significant decrease of mammosphere colony formation in suspension by 3-fold (Figure 4F). Importantly, *in vivo* tumorigenic analysis demonstrated that IWP2 was able to inhibit the tumorigenicity of HCT116 cells cultured with AMSCs. SCID mice subcutaneously injected with the IWP2 treated and AMSCs co-cultured HCT116 cells were found to develop smaller tumors in comparison with the untreated co-cultured cells (Figure 4G). Results from IHC staining further revealed that tumor samples generated with IWP2-treated AMSCs-co-cultured HCT116 cells expressed more abundant E-cadherin and less vimentin proteins, as compared with the untreated AMSCs-co-cultured HCT116 cells (Figure 4H). This result suggested a reduced malignancy of HCT116 when the Wnt signaling in co-cultured system was inhibited. Together, these results indicate that FGF10 and VEGFC may be candidate paracrine factors responsible for the malignancy of cancer cells induced by Wnt-activated AMSCs. Of note, a robust induction of FGF10 and VEGFC by AMSCs may require cancer cells with abundant activation of canonical Wnt signaling.

## Discussion

Interactions between MSCs and tumor cells involves a number of MSC secreted signaling molecules that may stimulate various signaling pathways, in particular related to cell growth and apoptosis regulation in the tumor cells. However, the crosstalk of tumor–MSC cells is complex, which involves in signaling from both of tumor cells and surrounding MSCs to control the tumor progression [20]. In this study, we show that cancer cells can secrete canonical Wnt signaling ligands (Wnt3a) to activate AMSCs to a TAF-like phenotype. As a consequence, the activated AMSCs can in turn produce FGF10 to induce CSC-traits of cancer cells. A crosstalk between the cancer cells and AMSCs thus may be essential to promote the tumor initiating of colon cancer cells and is associated with poor prognosis in colon cancer patients. In the primary tumor, cancer stem cell may arise from the transformation of resident stem cells or from dedifferentiation of differentiated tumor cells in response to specific microenvironmental signals [21]. It is therefore clear that the crosstalk between tumor cells and their surrounding microenvironment is required for CRC development. The delineation of key molecular pathways has enhanced our knowledge of the biology of tumor microenvironment, tumor dissemination, and carcinogenesis. The complexities of cell-cell communication and the possibilities for modulation provide new avenue for cancer treatments. However, the understanding of the dynamic regulation of relationships between cells in the tumor microenvironment was still indistinct.

During a progression of cancer development, cancer cells and the surrounding microenvironment constantly communicate each other through a biochemical and/or a biophysical cues. In this regard, signaling molecules in the microenvironment play an indispensible role in controlling cancer cells to undergo an EMT and promote the invasion and metastatic dissemination of cancers. In general, signaling molecules are not independent from each other, rather than interact to form a complex signaling network. In the present work, we found that AMSCs could induce EMT and enhance the invasive capability in colon cancer HCT116 cells. Epithelial colon cancer cells undergoing an EMT show mesenchymal features with loss of polarity and stem like spindle shape, which is closely associated with motility, metastasis and invasiveness. The EMT has also been connected to induction of cancer stem cells [22], drug resistance [23], and immune suppression. In this regards, many similar findings were also reported in varying models. Interestingly, we found that cell communication by a direct or an indirect manner resulted in distinct consequences, a direct co-culture of cancer cells with AMSCs showed a significantly enhanced potential malignancy of cancer cells, in comparison with the cancer cells cultured in the AMSCs conditional medium, as determined by the expression of stemness genes *Oct4*, *Nanog*, *Bmi1* and *Sox2*. The role of Bmi1 and Nanog in regulating stemness and drug resistance of breast cancer cells has been verified [24]. Thereby, we hypothesized that cancer cells might play a role in activating tumor-associated phenotypes of AMSCc, and in turn, the activated AMSCs could affect the ability of cancer cells through the paracrine signaling pathways. Indeed, extracellular signaling of FGF10, VEGFC, IL10 and TNFα of AMSCs was dramatically induced when they were co-cultured with cancer cells, and these molecules are suggested to have a direct or indirect impact on Wnt signaling pathway. This notion is supported by studies from others. For instance, Cohen et al. reported that Wnt3a could significantly increase the expression of FGF10 and a novel Wnt-FGF signaling axis was required for expansion of Isl-1–positive anterior heart field progenitors [25]. The finding of that Wnt-induced FGF-10 secreted by parabronchial SMC is essential for epithelial repair after naphthalene injury is another example [26].

Noteworthy, an increased expression of FGF-10, VEGFC, IL-10 and TNFα of AMSCs was found to be along with an elevated EMT gene expression of colon cancer cells in the co-culture model in this study. The important role of FGF-10 in type III EMT of cancer cells and the initiation of metastasis through various signaling pathways has also been suggested [27]. In the present study, we found that FGF10 was mainly responsible for the activation of AMSCs, which may in turn promotes the EMT of cancer cells to form the self-reinforcing loop. Accumulating studies suggest that ASCs like MSCs are capable of promoting angiogenesis through the secretion of growth factors, in particular VEGFC [28], since angiogenesis is a well-known crucial event for cancer growth, and VEGF secretion plays a pivotal role in this process. In addition, a novel Rspo1-Wnt-Vegfc-Vegfr3 signaling pathway has been defined to play an essential role in developmental angiogenesis [29], and an aberrant expression of VEGFC was recently found to be associated with lymph node metastasis in intrahepatic cholangiocarcinoma (IHCC) [30]. Here, we also found a decent increase of VEGFC in AMSCs, suggesting that VEGFC secreted by AMSCs may be in part attributed the increased expression of EMT gene in colon cancer cells.

In addition to the FGF10 and VEGFC, the expression of inflammatory factors IL-10, INFγ and TNFα was also increased in co-cultured AMSCs. Inflammations have long been recognized to be associated with cancer development, in which cancer cells can recruit activated fibroblasts and immune cells that in turn secrete varied cytokines to induce cancer development and metastasis in part by directly regulating the EMT program [31,32]. In this context, several recent clinical studies showed that IL-10 was an immune suppressor of different cancers that able to lead an adverse outcome in terms of immune tolerance [33,34]. In addition, an activation of beta-catenin signaling can program dendritic cells to a tolerogenic state, and limits the inflammatory response by IL-10 [35]. Moreover, Razmkhah *et al.* recently found that resident adipose stem cells (ASCs) in breast cancer tissue might play crucial roles in breast tumor growth and progression by inducing regulatory molecules and promoting anti-inflammatory reaction within the tumor microenvironment enriched in IL-10 and TGF-β1 [36]. An up-regulated IL-10 was also found in AMSCs co-cultured with colon cancer cells in this study. Apart from inflammatory cytokines, MMPs in the tumor stroma are also important elements that play key roles in the matrix remodeling, inflammation, angiogenesis, cell growth and migration of cancers, in either a proteolytic or a nonproteolytic manner [37]. Indeed, a previous study using a co-culture model of breast cancer cells and bone marrow MSCs showed that cancer cells could induce a up-regulated expression of MMPs in MSCs, along with elevated expression of EMT related genes in cancer cells [38].

The role of MSC signaling through Wnt in cancer progression has been reported in previous publications, despite an inhibitory effect on tumor growth was reported in a study using Z3-MSCs, a cell line producing Dkk-1, an inhibitor of Wnt/β-catenin signaling [20,39]. In this study, we assume that an activation of Wnt signaling is an effect of AMSCs response to Wnt signaling hyperactivated cancer cell lines that aberrantly expressing Wnt ligands, such as the Wnt3a, an essential component in the Wnt signaling pathway. Indeed, the Wnt signaling of AMSCs was activated when they were co-cultured with cancer cells; sequentially the Wnt-activated AMSCs increased their expressions of EMT genes, including *FGF10*, *VEGFC*, *MMPs*, and cytokines. These signaling molecules in turn positively activate Wnt signaling of cancer cells to form a positive paracrine factor feedback loop, which leads to an uncontrolled cell growth, proliferation and invasion of cancer cells.

## Conclusions

In summary, this study demonstrates an important role of the crosstalk between colon cancer cells and AMSCs in in the metastatic potential of colon cancer cells. The paracrine factors of stroma cells in cancer microenvironment are essential for cancer cells to maintain or promote their capacity of invasion and migration, which could be a potential therapeutic target for colon cancer metastasis. In this context, the CSCs are regulated by complex interactions with the components of the tumor microenvironment including mesenchymal stem cells, through microenvironmental networks of cytokines and growth factors. Since these components have a direct effect on metastatic properties of cancer cells, they represent attractive therapeutic targets for colon cancer treatment.

## Abbreviations

Wnt: Wingless-type MMTV integration site family
AMSCs: Adipose mesenchymal stem cells
MMPs: Matrix metalloproteinases
FGF: Fibroblast growth factor
VEGF: Vascular endothelial growth factor
IL: Interleukin
TNF: Tumor Necrosis Factor
IGF: Insulin-like growth factor-1
TGF: Transforming growth factor
HGF: Hepatocyte growth factor
